# Perceptions of Firearm Accessibility and Suicide Among US Adults Living in Households With Firearms

**DOI:** 10.1001/jamanetworkopen.2022.39278

**Published:** 2022-10-31

**Authors:** Andrew Conner, Deborah Azrael, Matthew Miller

**Affiliations:** 1 Digestive Disease & Surgery Institute, Cleveland Clinic, Cleveland, Ohio; 2 Harvard Injury Control Research Center, Harvard T. H. Chan School of Public Health, Boston, Massachusetts; 3 Bouvé College of Health Sciences, Northeastern University, Boston, Massachusetts

## Abstract

This survey study of US adults living in households with firearms uses a counterfactual question to assess whether respondents believed restricting access to firearms might prevent death by suicide.

## Introduction

In 2020, 45 979 people died by suicide in the US; half used firearms.^1^ We identified studies on how US adults think about the association between household guns and suicide.^2,3^ One study reported that 46% of emergency department clinicians believed that most people who die by suicide using firearms would have found another way to die by suicide had the firearm not been accessible, and 9% believed that no lives would have been saved.^2^ Another study noted that fewer than 10% of US adults living in a household with firearms agreed that “the presence of a firearm in the home increases the risk for suicide.”^3^ In a survey of US adults living in households with firearms, we used a counterfactual question to assess whether respondents believed restricting access to firearms might prevent death by suicide.

## Methods

A probability-based online survey was conducted from July 30 to August 11, 2019.^4^ Data analysis was performed from September 29, 2021, to July 25, 2022. The sample included adults living in homes with firearms. Race and ethnicity data were included because perceptions about the effect of firearm accessibility on suicide appear to differ by race and ethnicity. The primary outcome was based on the question “Each month in the US, approximately 2000 people die by suicide using a firearm. If a firearm had not been accessible to them, what percentage of these 2000 people (that is, how many out of every 100) do you think would have found another way to kill themselves?” Responses ranged from 0% to 100%, dichotomized as all (100%) vs not all (<100%). We plotted nondichotomized responses as frequency distributions, stratified by personal firearm ownership status.

Analyses used the SVY suite in Stata IC, version 16 (StataCorp LLC), weighted to generate national estimates. We followed the AAPOR reporting guideline for survey studies. The institutional review boards at Northeastern University and Harvard School of Public Health determined the survey did not require review because the deidentified data did not qualify as human participant research.

## Results

A total of 4379 of 6721 English-speaking adults (65.2%) responded to the survey invitation; 4030 (92.0%) completed the survey and 3925 responded to our key question. Compared with respondents who provided an answer to this question, respondents who refused to answer the question were significantly more likely to be younger (33.5%; 95% CI, 31.9%-35.2% of respondents who answered our primary question were aged ≥60 years vs 20.4%; 95% CI, 13.9%-29.0%) of those who did not respond to the primary question.

A total of 2122 respondents (54.1%) were men, 32.3% (95% CI, 30.4%-34.2%) lived with a child (age <18 years), and 67.3% (95% CI, 65.4%-69.1%) owned a firearm (Table). Thirty-six percent (95% CI, 34.2%-37.8%) believed that eliminating access to firearms would not have saved lives; this belief was shared by 41.1% (95% CI, 39.0%-43.4%) of firearm owners and 25.3% (95% CI, 22.4%-28.4%) of adults living with gun owners (Table). An additional 46.9% (95% CI, 44.7%-49.1%) of firearm owners and 55.5% (95% CI, 51.9%-59.0%) of nonfirearm owners indicated that more than 50% of persons who attempted suicide would have found other means (Figure).

**Table.** Proportion of US Adults Who Believe That Reducing Access to Firearms Would Have No Effect on Suicide Rates^a^

Characteristic	Respondents, No. (%)^b^	Firearm ownership status, % (95% CI)		
		Firearm owner	Nonowner who lives with owner	Total
All respondents	3925 (100)	41.1 (39.0-43.4)	25.3 (22.4-28.4)	36.0 (34.2-37.8)
Sex				
Female	1803 (48.0)	43.3 (39.3-47.2)	26.5 (23.4-29.8)	33.6 (31.2-36.2)
Male	2122 (52.0)	40.2 (37.6-42.9)	18.9 (11.9-28.9)	38.1 (35.6-40.7)
Age group, y				
18-29	271 (15.8)	35.6 (27.6-44.5)	20.5 (13.9-29.2)	28.4 (22.9-34.6)
30-44	746 (22.5)	39.6 (34.8-44.5)	28.4 (22.4-35.2)	35.9 (32.1-40.0)
45-59	1124 (28.2)	45.4 (41.5-49.3)	28.5 (23.0-34.8)	40.6 (37.4-44.0)
>60	1784 (33.5)	40.5 (37.5-43.5)	24.1 (19.8-29.0)	35.6 (33.1-38.2)
Race and ethnicity				
Non-Hispanic White	3279 (76.8)	42.7 (40.4-45.1)	24.9 (21.8-28.4)	37.0 (35.0-39.0)
Non-Hispanic Black	227 (7.7)	21.6 (15.4-29.3)	22.9 (13.2-36.7)	22.0 (16.5-28.6)
Non-Hispanic other^c^	175 (5.4)	40.5 (29.6-52.4)	27.9 (16.2-43.7)	36.1 (27.4-45.7)
Hispanic	244 (10.1)	45.3 (36.8-54.1)	27.8 (17.9-40.3)	38.7 (32.0-45.9)
Children in the home, y				
None aged <18	2946 (67.7)	39.9 (37.4-42.4)	24.8 (21.5-28.5)	35.2 (33.2-37.3)
Any aged <18	979 (32.3)	44.0 (39.7-48.4)	26.1 (20.9-32.2)	37.5 (34.0-41.1)
Educational level				
Less than bachelor's degree	2280 (69.1)	45.0 (42.1-47.8)	26.7 (23.0-30.8)	38.8 (36.5-41.2)
Bachelor's degree or higher	1645 (30.9)	33.0 (30.0-36.2)	21.8 (17.7-26.5)	29.6 (27.1-32.2)
Lives in a metropolitan statistical area^d^				
No	782 (20.5)	49.4 (44.5-54.4)	29.5 (22.6-37.5)	42.8 (38.6-47.1)
Yes	3143 (79.5)	39.0 (36.6-41.5)	24.2 (21.0-27.6)	34.2 (32.2-36.2)
US region				
Northeast	492 (11.8)	42.9 (36.9-49.1)	28.9 (20.8-38.6)	37.8 (32.8-43.0)
Midwest	1014 (23.6)	40.1 (35.8-44.5)	24.9 (19.6-31.0)	34.6 (31.2-38.2)
South	1534 (42.0)	41.8 (38.4-45.3)	24.5 (19.9-29.7)	36.5 (33.7-39.5)
West	885 (22.5)	40.1 (35.6-44.7)	25.1 (19.2-32.1)	35.4 (31.7-39.2)

^a^ Based on responding that every 1 of the approximately 2000 people who die by suicide with a firearm every month would have found another way to kill themselves had the firearm not been accessible to them. A total of 105 respondents refused to answer the question regarding what proportion of suicide decedents would have found another way to kill themselves had a firearm not been accessible.

^b^ Percentages reported are weighted percentages meant to be representative of US population. Poststratification weights were applied to adjust for nonresponse and undercoverage or overcoverage from the study-specific sample design relative to expected distributions from the US Census Current Population Survey and the American Community Survey.

^c^ This designation includes people who do not identify as Hispanic and identify as one of the following categories: American Indian or Alaska Native, Asian, Native Hawaiian or other Pacific Islander, or a different race. It may also include other groups who do not identify as Black or White.

^d^ A core area containing a substantial population nucleus, together with adjacent communities having a high degree of economic and social integration with that core.

**Figure.**
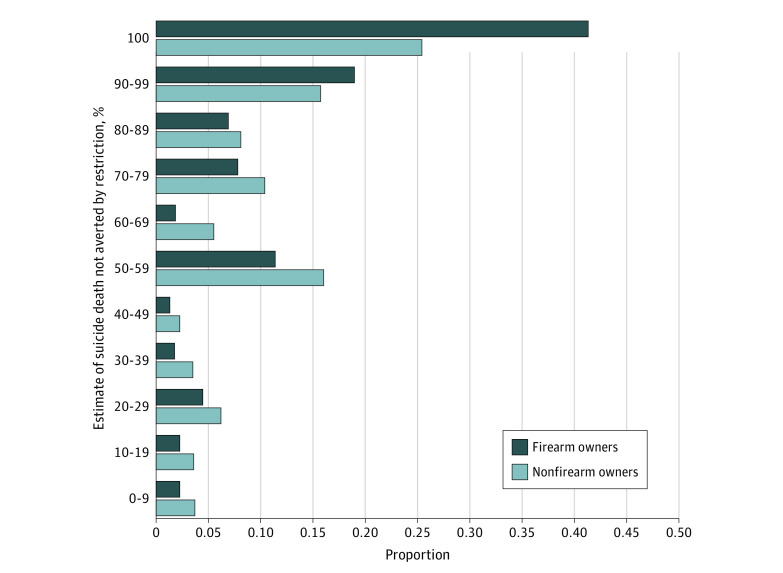
Respondent Estimates of the Proportion of Suicide Death Not Averted by Restricting Access to Firearms, by Personal Firearm Ownership Status

## Discussion

Consistent with studies reporting skepticism among US adults regarding suicide prevention efforts that rely on restricting access to highly lethal and commonly used methods,^2,3,5,6^ we found that 1 in 3 adults living in households with firearms believed that restricting access would not prevent any deaths by suicide; most believed that more than half of those who used a firearm would find equally lethal means.

A limitation of our study is that the primary outcome was based on a single question regarding a counterfactual scenario. Our results are also subject to potential inaccuracies due to social desirability bias and the possibility that respondents who chose not to participate may have differed in ways related to their beliefs vs those who participated.
